# Assessment of COVID-19 Waste Flows During the Emergency State in Romania and Related Public Health and Environmental Concerns

**DOI:** 10.3390/ijerph17155439

**Published:** 2020-07-28

**Authors:** Florin-Constantin Mihai

**Affiliations:** Department of Research, Faculty of Geography and Geology, Alexandru Ioan Cuza University, Carol I Blvd, Nr.20 A, RO-700505 Iasi, Romania; mihai.florinconstantin@gmail.com

**Keywords:** COVID-19, waste management, medical waste, municipal waste, public health, pollution

## Abstract

This paper provides a rapid assessment method of potentially infectious waste flow related to the coronavirus disease (COVID-19) pandemic in Romania focusing on the emergency state (from 16 March to 14 May 2020) where a national lockdown was in force with restrictive and social distancing measures concerning population mobility and economic activities. Medical and municipal waste management systems are critical services in combating the virus spread in the community. This assessment is useful due to poor available data of medical waste flow in environmental reports and it covers COVID-19 patients, quarantined, and self-isolated persons as the main potential infectious waste sources. The proposed model estimates that COVID-19 related waste flow is 4312 t at the national level from 25 February to 15 June of which 2633 t in the emergency state period. This assessment is correlated with deficiencies of medical and municipal waste management systems in Romania before the COVID-19 pandemic as stress factors of public health and environment. This study points out the main challenges of waste operators and reveals some best practices during this pandemic crisis. Based on the results and discussion section, several recommendations are proposed to COVID-19 waste-related issues and points out the crucial role of the reliable medical and municipal waste database in managing such biologic hazards at national and EU levels. Monitoring of COVID-19 waste flow through such models are important for decision-makers, particularly in low and middle-income countries which are facing waste management deficiencies and gaps in waste statistics, to reduce other contamination risks or related environmental threats.

## 1. Introduction

The geographic expansion of the COVID-19 outbreak around the world forced the World Health Organization (WHO) to declare the pandemic status on 11 March 2020. On 24 March, the United Nations Environment Programme (UNEP) pointed out that waste management is an essential public service in avoiding secondary impacts upon public health and environment and special attention should be paid to handle medical, household and other hazardous items in such a period [1]. Massive amounts of medical waste have already been generated and disposed into the natural environment [2] and waste mismanagement practices can increase the contamination risks in developing countries [3,4] or among urban poor which live in informal settlements [5,6] or among hundreds of millions of people without basic water, sanitation, and waste management facilities in rural areas [7].

Romania declared a state of emergency on 16 March due to the pandemic crisis in which a national lockdown was implemented since the first case of COVID-19 patient was registered on 26 February 2020 [8]. The National Public Health Institute of Romania declared household waste generated in quarantines as infectious waste on 18 March 2020. Therefore, there is a shift from usual municipal waste management practices towards the special collection, transport and disposal requirements of hazardous wastes [9]. On 30 March, the European Center for Disease Prevention and Control (ECDPC) released an infection prevention and control guideline at the household level for suspected or confirmed COVID-19 disease with mild symptoms in self-isolation status [10]. This document provides some recommendations regarding the safe management of household waste. One keynote is to have a double waste bag for used tissues, face masks and other waste, which should be disposed of in the residual bin. The European Commission (EC) released a guideline on 14 April regarding waste management practices in the context of coronavirus crisis [11]. This document requires to maintain quality standards of municipal waste collection services and medical waste treatment and any measures must comply with EU regulations. Zero Waste Europe (ZWE) argues that the COVID-19 crisis should not undermine the EU’s long-term circular economy objectives by returning to business as usual [12]. The transition towards a circular economy should be further supported while 2020 is the first year where the recycling rate of 50% should be achieved by the EU Member States. However, this target is impossible to be achieved in Romania where the recycling rate of municipal waste was around 14% in 2017 [13].

Romania is coping with several mismanagement practices such as poor efficiency of source-separated waste collection schemes in urban areas, the prevalence of landfills as a main waste management option, delays in implementation of regional integrated waste management systems, lack of a reliable waste statistics database, etc. Furthermore, rural Romania is facing illegal waste disposal practices (wild dumps, open burning practices), plastic pollution of freshwater bodies and limited waste collection coverage of the rural population in some counties [14,15]. The circular economy remains underdeveloped despite the promising potential in this area [16], but the new circular economy plan promoted EU could catalyze the effort in the right direction [17]. On the other side, medical waste management issues are less examined in Romania, poor details and waste statistics data provided in environmental reports and several environmental crimes investigated by environmental authorities and revealed to the general public through national and local mass-media with a particular focus to hazardous waste incinerators. Neither the new national waste management plans provide a solid and updated analysis of medical waste management status in Romania [18]. According to WHO, about 85% of medical waste generated by healthcare activities is nonhazardous and the remaining 15% is considered hazardous material that can be infectious, toxic or radioactive [19]. However, COVID-19 related waste flow is considered infectious waste which must be properly treated [20,21], but serious concerns are raised around consumption and management of plastic products associated with personal protective equipment (PPE) [22,23]. A weak infectious waste management system could speed up the growth of COVID-19 in developing countries [24].

On this background, this paper aims: (i) to provide a rapid assessment of potentially infectious waste broken down in three main sources such as COVID-19 patients (hospitalized), people in quarantined sites (abroad working/travel Romanian citizens from countries/regions heavily affected by coronavirus), self-isolated people (persons from abroad or contacts with confirmed COVID-19 cases); (ii) to reveal national and some regional flows of potentially infectious waste generated; (iii) to identify critical issues during the emergency state in Romania related to medical and municipal waste management backgrounds; (iv) to reveal some best practices related to waste management sector; (v) to provide some recommendations related to waste management policies and data support at national and EU levels.

This study intends to provide a rapid assessment method to estimate COVID-19 related waste flow in Central and Eastern European countries where medical and municipal waste management systems must be further improved to comply with EU standards. Furthermore, such analysis is useful in case of low and middle-income countries around the world which face poor waste management infrastructure and gaps in waste statistics data, as an alternative solution to estimate the potential COVID-19 related waste flow necessary for decision-makers to reduce other contamination risk routes and environmental pollution threats.

## 2. Materials and Methods

### 2.1. COVID-19 Status in Romania

The first case of COVID-19 patient waste detected on 26 February 2020 increasing to 168 cases on 16 March (0 deaths and 9 recovered) when the state of emergency was implemented. The first deaths were registered one week later (9 on 23 March). After the first month of emergency state (15 April) the COVID-19 statistics revealed 7216 (cumulative cases), 419 deaths, 1270 recovered and at the end of the emergency state (and beginning of alert state on 15 May) the situation was 16,247 cumulative cases of which 6020 active cases, 1090 deaths and 9370 recovered. By the end of the first stage of the alert state (15 June) statistics show 22,165 cumulative cases of which 4938 active cases, 1410 deaths and 15,817 recovered.

The peak of active cases was recorded in the second stage of emergency state with over 7000 cases from 23 April to 13 May, dropping during the alert state under 5000 cases from 28 May to 15 June. The number of patients in intensive care units (ICU) increased since 19 March (6 cases) to the peak of 288 cases (22 April), maintain the threshold of plus 200 cases until the end of the emergency state (15 May). However, the cases of intensive care patients decreased in the alert state below 200 cases until 15 June. The number of persons in quarantine has increased since 15 March (2855) to the peak on 9 April (25,586) dropping to 11,631 (28 April) with a slight increase by the end of emergency state 14,441 (15 May) followed by a continuous decreasing trend during the alert state (1331 on 15 June). The number of persons self-isolated increase from 14 March (14,680) to the peak on 29 March (132,641) with a continuous descending trend by the end of emergency state such as 14,789 (15 May). However, there is an ascending trend during the alert state from 19 May (24,415) peaking by the end of month 98,403 (30 May) and maintaining this trend in June surpassing 100,000 persons on 11 June than declining by 15 June (92,734 persons).

These statistics are important in the assessment process of Covid-19 related waste flow in Romania as further described in the following section.

### 2.2. Estimation of COVID-19 Waste Flow in Healthcare Facilities

High-income countries generate on average up to 0.5 kg of hazardous waste per hospital bed per day while low-income countries generate on average 0.2 kg [19]. On the other side, there are no separate collections (hazardous vs nonhazardous items) in developing countries that increase the ratio of hazardous items through contamination [25]. At the national level, medical waste as follows:

M_w_ = No. of active COVID-19 cases per day × M_wgr_ (kg∙bed∙day^−1^) (1)

where M_wgr_ = medical waste generation rate—1 kg∙bed∙day^−1^ The number of active cases is available at https://www.graphs.ro/ at the national level. Active cases represent confirmed COVID-19 patients that are treated in hospitals without recovered or dead patients. In general, all COVID-19 cases were treated in hospitals during the emergency state.

Subnational level (county): Mw = confirmed COVID-19 cases per day × Mwgr (kg∙bed∙day^−1^). (2)

There is no available information about the active COVID-19 statistics at the county level and some inconsistencies could appear between confirmed cases, deaths, and recovered people at this scale. However, daily data provided by the strategic communication group of the Romanian government are considered for estimation to reduce this aspect. Therefore, COVID-19 medical waste flow is calculated from active cases at national level, whereas subnational is calculated from daily confirmed cases.

The assessment of healthcare waste at subnational levels is more difficult to achieve, the reporting systems are made by regional public health authorities (county departments) to the National Institute of Public Health. However, national authorities stopped reporting data at county levels from 19 March until 2 April. On 20 March, a manifesto initiated by Geo-spatial.org and supported by 17 non-governmental organizations (NGOs) was addressed to national authorities to release such key daily COVID-19 statistics data at county level (including confirmed cases, recovered, deaths, no. of people in quarantine or self-isolated and details about age and gender in each category for data consistency, no. of tests performed each day) for better monitoring of COVID-19 status in Romania and to avoid misinformation and spreading of fake news [26].

The average medical waste generation rate was around 1 kg∙bed∙day^−1^ in Infectious Diseases Clinical Hospital of Craiova used as the baseline for this paper [27]. This value is consistent with the recommendation of El-Haggar [28] as the average amount of medical waste generated per bed per day.

The highest rate of medical waste 1.814 kg∙bed^−1^∙day^−1^ was calculated in the case of the Emergency Clinical Hospital of Craiova [29]. The medical waste generation rates in main hospitals vary between 1–1.8 kg∙bed^−1^∙day^−1^ compared to smaller healthcare facilities < 0.5 kg∙bed^−1^·day^−1^ [27,29].

In Italy, Vaccari et al. [30] found that the highest medical waste generation in departments of anesthetics (5.96 kg∙bed^−1^∙day^−1^) pediatric and intensive care (3.37 kg∙bed^−1^∙day^−1^). In Wuhan (China) the amount of medical waste peaked to 240 t per day [24]. Therefore, in the COVID-19 pandemic context, medical waste flow associated with intensive care patients is expected to be higher than the general average of infectious disease and upper limited detected by previous studies in Romania (1.8 kg) is further considered in the analysis.

M_w_ = No. of COVID-19 patients in ICU. day^−1^ × 1.8 kg∙bed^−1^∙day^−1^ (3)

The ICU waste is included in the general COVID-19 medical waste flow as calculated by Equation (1).

### 2.3. Estimation of Potentially Infectious Waste Generated in Quarantine Places or by Self-Isolated People in Households

Quarantine (14 days) is established for all people who have no symptoms but returning from areas with extended community transmission of new coronavirus (COVID-19 red areas) in special locations (e.g., hotels, touristic pensions) supervised by local authorities and county departments of public health. In the first phase, these locations were served by municipal waste operators, but national authorities considered these wastes as infectious. In this situation, the quarantine places must have contracts with special waste operators licensed to collect and transport such hazardous wastes to waste incinerators.

Self-isolation (14 days) is established for people who do not show symptoms, but: (i) have traveled in the last 14 days to regions/localities in areas affected by COVID-19 other than those with extended community transmission (yellow areas); (ii) direct contact with people with symptoms and who traveled to areas with extended community transmission; (iii) direct contact with people who were confirmed with coronavirus (COVID-19); (iv) family member in above-mentioned cases. These waste generated in such households should be disposed of in residual bins and collected by waste operators and transported to landfills.

The estimation of waste flow data takes into consideration the municipal waste generated in households or quarantine places.

In Romania, municipal waste generation rate is 0.64 kg∙inhab∙day^−1^ in urban areas and 0.31 kg∙inhab∙day^−1^ in rural areas according to the new national waste management plan [18].However, regional differences are expected between larger cities (e.g., county capitals) and the other urban areas.

The regional waste management plans of Dolj county points out that Craiova city has a per-capita waste generation rate of 0.7 kg∙inhab∙day^−1^ compared to other cities 0.55 kg∙inhab∙day^−1.^ [31]. In the latter case, this per-capita generation rate is used as a conservative option to estimate the municipal waste flow in quarantine places and self-isolated cases in households.

Quarantine/self-isolation waste = no. of people in quarantine/self-isolated (national & subnational levels) × W_GR_, W_GR_ = waste generation rate (0.55 kg∙inhab∙day^−1^). (4)

**Figure 1:**
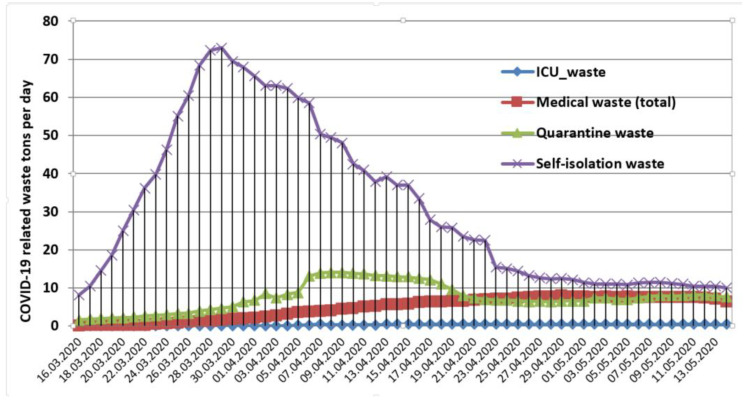
COVID-19 related waste flow in Romania during the emergency state.

Regional assessments of such wastes depend on available data at county levels regarding the number of persons in quarantine or self-isolated. These data are less available to the public than confirmed cases at the county levels. At the beginning of April, all three main categories (confirmed, quarantine, self-isolated) were available for a short time (see Figure 1). The number of quarantine and isolated are daily provided by the press release notes at the national level (https://stirioficiale.ro/informatii) by the strategic communication group of the Romanian government. Some data are available for certain counties through government representatives at county levels (Prefecture Institution), but the lack of cohesion across all counties reduce the possibility to perform a spatial analysis during all emergency period at subnational levels. In this context, a snapshot of regional disparities is presented for the beginning of April (all three sources of COVID-19 related waste flow) and completed with certain case studies (e.g., Neamt County).

Plastic packaging materials are significant waste streams generated in quarantine places due to the single-use of cups, tableware and dishes according to three daily meals. An increase in plastic and glass was observed in Turin (Italy), but that overall MSW production in March 2020 was 11.5% lower than the one registered in March 2019 [32]. In addition, plastic waste increased in Thailand, but in Indonesia the amounts of waste sent to landfills decreased up to 40% due to the closure of offices, restaurants and industries [24].

### 2.4. Limitations of the Study

The paper aims to provide a rapid estimation of potentially infectious waste related to coronavirus in Romania at national and subnational levels in the most critical period (emergency state 16 March to 15 May) and the first stage of the alert state (15 May to 15 June). This assessment depends on the reliability of COVID-19 statistics in Romania and the transparency of local and national authorities in this regard as mentioned in Section 2.2 and Section 2.3 (number of confirmed cases, active cases, number of population in quarantine/self-isolate in homes, number of tests, etc.) and on the other hand, the medical waste generation rate (kg∙bed∙day^−1^) and municipal waste generation rates are taken into consideration and supported by previous studies. Some inconsistences between the reported cases from one county to another could be expected, but daily official statistics are taking into account. In the medium term, experimental studies at various COVID-19 hospitals and quarantined places should be made to adjust waste generation rates and to determine the medical waste composition/municipal waste composition. Another possible objective is to determine the medical waste generation rate broken down per intensive care patients and non-intensive care patients. Such field measurements are necessary for Romania and beyond to update the baseline scenario in both healthcare facilities and households and ultimately to improve the current models. However, special attention should be given in the international comparison of COVID-19 pandemic data due to various reporting systems and the number of tests performed [33].

## 3. Results and Discussions

### 3.1. COVID-19 Waste Flow at National Level

Table 1 shows that most of the COVID-19 related waste was generated during the emergency state where medical waste (COVID-19 patients) has a ratio of 10.86% and quarantine waste 17.22% of the total waste flow.

These two sources cumulated 739.48 t of waste which should be incinerated according to national authority guidelines adding other 262 t during the alert state. Per the total period 1007.7 t infectious wastes (healthcare facilities + quarantine wastes) should have been collected by special hazardous waste operators and disposed of in the waste incineration plants or using alternative options for medical waste flow as outlined in Section 3.4.

For example, in Jakarta (Indonesia), the amount of medical waste reached 12,740 t 60 days after the virus attacked the region and in Wuhan (China) 5200 t of medical waste was collected from individual containers which served hospitals, isolation areas and shelters [24].

Self-isolation wastes prevailed in all periods as amounts generated, but this waste stream has a lower infectious potential being managed by ordinary municipal waste operators and disposed of in landfills. However, some local authorities treat the self-isolation wastes as quarantine wastes with special transport and disposal treatment.

The ICU patients generated around 6% of total medical waste during the emergency and the alert state states (16 March to 15 June) with no such waste stream before the emergency state. Prior the emergency state, the amounts of potentially infectious waste flow was over 10 times smaller than both emergency and alert states which point out the exponential growth of COVID-19 cases and on the other hand, the large number of people who returned in Romania from countries seriously affected by COVID-19 pandemics such as Italy or Spain.

National Institute of Public Health (NIPH) updated the list of red and yellow areas destinations in which quarantine and self-isolation are compulsory when citizens return in Romania which was regularly updated during the emergency state [34]. The list of extended community transmission comprised on 26 February (first COVID-19 case detected in Romania): Mainland China (Hubei Province), Wenzhou, Hangzhou, Ningbo, Taizhou Cities in Zhejiang Province or one of the 12 localities in Italy (Lombardia and Veneto regions). The list comprises on 15 March 2020 (beginning of emergency state) in (i) red areas (quarantine): Hubei region (China), Italy, Iran, South Korea (Daegu city and Cheongdo County); (ii) yellow areas (self-isolation)—countries with over 500 cases (Austria, Belgium, South Korea, Denmark, Switzerland, France, Germany, Japan, UK, Norway, Netherlands, USA, Spain, Sweden, rest of China. At the end of the emergency state (15 May), the self-isolation is compulsory for all international travels (including family members in the same household). To avoid exposure of family members the traveling person can require to be self-isolated in special quarantine places supervised by authorities.

**Figure 2:**
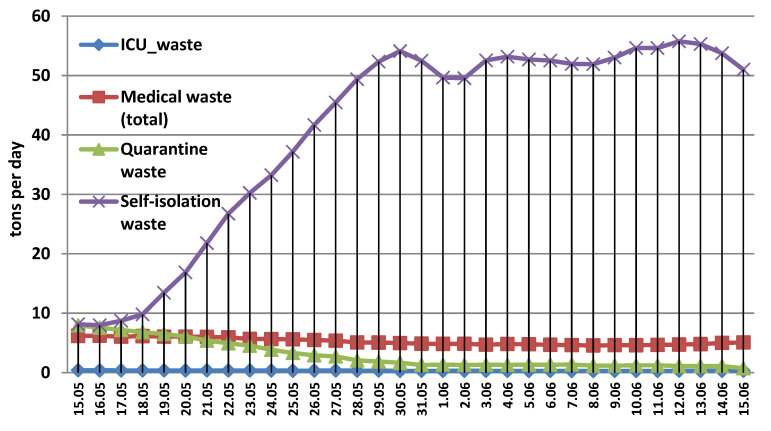
COVID-19 related waste flow in Romania during the alert state.

During the emergency state, the model estimates that the self-isolation waste flow peaked 28 and 29 March over 70 t per day surpassing the threshold of 50 t per day from 25 March to 7 April followed by a descending trend where the daily amounts dropped around 10 t as shown in Figure 1. However, this waste stream regained an ascendant trend during the alert state reaching the threshold of 50 t per day from 26 May to 15 June as shown in Figure 2.

In both periods, the self-isolation waste is the largest COVID-19 related waste flow, but without severe regulations in their collection and treatment practices as the wastes generated by healthcare facilities or quarantine places. Medical waste stream comprised the ITU patients and those with mild symptoms treated in dedicated hospitals. It is estimated that ITU patients generated an average of 0.286 t per day during the emergency state peaking on 22 April (0.548 t) then it dropped to around 0.2–0.3.

The wastes generated by confirmed COVID-19 patients from healthcare facilities are around 4.8 t per day on average during the emergency state peaking on 29 April (7.91 t). However, this peak was not reached in the alert state (15 May to 15 June), but the average was slightly higher around 5.23 t per day. A key aspect is that medical waste flow is larger than quarantine flow from 22 May until the end of the alert state on 15 June. The daily average of quarantine waste is around 3 t per day during the alert state compared to 7.55 t per day during the emergency period. In the latter case, quarantine wastes reached the threshold of 10 t from 6 April to 18 April peaking to 14 t on both 8–9 April. Per total COVID-19 waste flow (medical + quarantine + self-isolation) the daily average is around 44 t per day during the emergency state and around 49 t during the alert state. The total maxim value of COVID-19 related waste flow reached 79.29 t on 29 March (emergency state) and 61.6 t on 12 June (alert state). In the next section, a regional analysis of COVID-19 flow is performed.

### 3.2. COVID-19 Waste Flow at Subnational Levels

**Figure 3:**
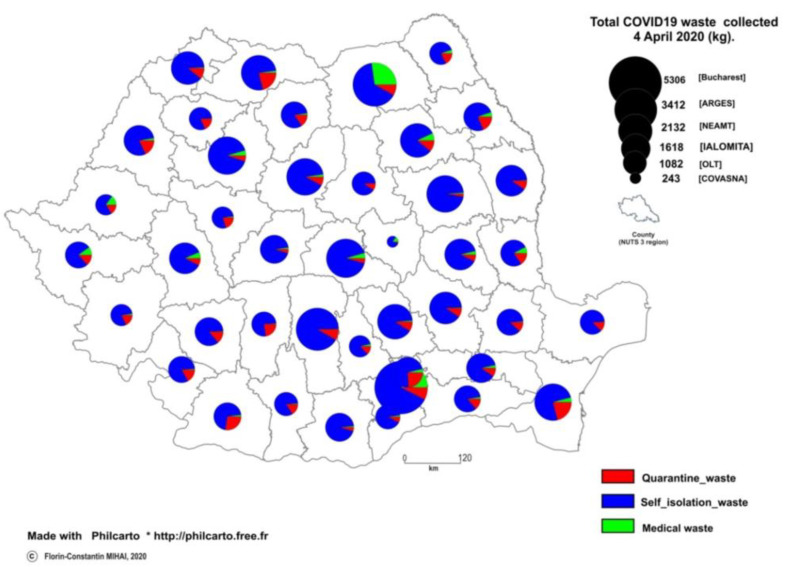
Regional disparities in COVID19-related waste flows as of 4 April 2020 (emergency state) Counties info: https://en.wikipedia.org/wiki/Counties_of_Romania.

As mentioned to the material and method section, the COVID-19 related data at subnational levels (county data) is difficult to obtain during the emergency state, particularly for quarantine or self-isolation persons. Figure 3 provides a regional snapshot of total potential infectious waste related to COVID-19 in Romania as of 4 April 2020. The map shows regional disparities of such waste flows with a prevalence of Bucharest capital city compared to other counties.

Total potential infectious waste is 73,870.7 kg of which 3386 kg medical waste (COVID-19 patients) and 8566.8 kg quarantine waste. These two waste streams (11,942.8 kg) should be collected by special waste operators and incinerated according to authorities’ recommendations. The rest of the 61,917.9 kg. is self-isolation waste. The medical waste (green section of the pie chart) is the most hazardous flow with larger amounts in Suceava County (967 kg), Bucharest (550 kg), Neamt County (148 kg), Timis County (136 kg), Brasov County (127 kg), Cluj County (121 kg). The lowest values are calculated in the case of Harghita (1 kg), Salaj (5 kg), Valcea (9 kg), etc.

Most quarantine wastes (red section of the pie chart) are generated in Constanta County (539 kg), 400–410 kg (Bucharest, Dolj, Ilfov) which are covered by waste incineration plants. At that time few quarantine wastes (<100 kg) were generated in Hunedoara, Sibiu, Covasna and Harghita counties. Self-isolation wastes are mostly generated in Bucharest city (4354 kg) followed by Arges (3166 kg), Brasov (2607 kg), Mures (2300 kg) which drop below 1000 kg (Dambovita, Valcea, Caras-Severin, Alba, Arad, etc.) lowest values in all three waste categories being in Covasna County.

Three are several counties where medical and quarantine wastes are at the lowest levels compared to self-isolation wastes (e.g., Tulcea, Vaslui, Gorj, etc.) which decrease the contamination risk of spreading COVID-19 disease by infectious waste.

**Figure 4:**
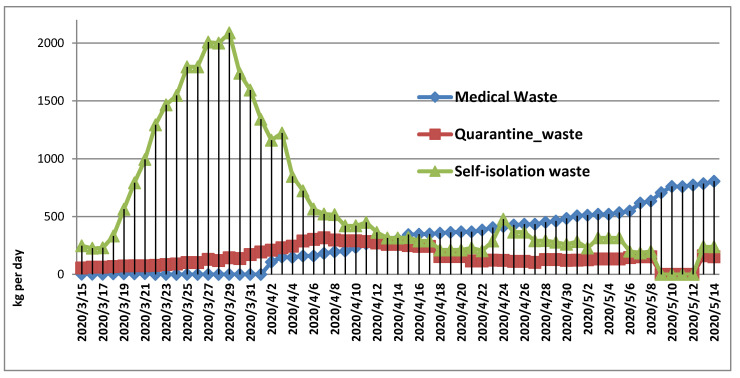
COVID-19 related waste flow in Neamt County during the emergency state.

In the case of the Neamt County, the model suggest that medical wastes generated by COVID-19 patients were increasing toward the end of the emergency state as the virus spreading in this area peaking in the last day to 805 kg. On the opposite side, the amounts of self-isolation wastes peaked in the first stage of the emergency state (27–29 March) surpassing 2000 kg of waste per day. The descend trend started by the end of March and the first week of April when the amounts of waste dropped below 500 kg per day until the end of the emergency state (14 May). The high number of self-isolation persons is explained by the large number of people who worked abroad and returned from the yellow areas of Italy or Spain. Fewer persons were quarantined (red zones) which generated fewer wastes (9160 kg) compared to self-isolation persons (37,000 kg). However, quarantine wastes are considered infectious waste which must be transported by special waste operators towards waste incineration plants as a medical waste of COVID-19 patients. In the latter case, it is estimated that the total amount generated during the emergency is almost twice (18,090 kg) than quarantine wastes as cumulative cases are still increasing during the alert state. Infectious wastes (medical + quarantine) reached 27,250.8 kg of total 64,256 kg COVID-19 related waste flow during the emergency state. There is a gap of data between 9 May to 12 May regarding the number of people in quarantine and self-isolation as shown in Figure 4 therefore, the total COVID-19 related flow is difficult to estimate at subnational levels. In the COVID-19 context, waste-related data are scarce and fragmented, and this situation is similar in other countries [24,32]. At the national level, the amounts of quarantine wastes are larger than healthcare wastes during the emergency state, but this situation is changing during the alert state as shown in Table 1.

### 3.3. Public Health and Environmental Concerns

In the first weeks of COVID-19 cases in Romania, the wastes collected from quarantine places were handled by municipal waste operators and disposed into landfills. Because such wastes were declared by national authorities as infectious wastes (first notification on 18 March 2020) the regional public health departments supervise the waste collection process between these sites and special waste operators licensed to transport hazardous waste to disposal facilities (e.g., hazardous waste incinerators). However, these wastes operators do not have wide geographic coverage and in times of emergency had difficulties to fulfill the requests from hospitals and quarantine sites. As an example, piles of potentially infectious waste bags formed in front of a quarantine site in Galati city because there is only one waste operator authorized to transport such wastes in the South-East Region. Mass media reveals cases where full bins and piles of medical wastes (outdoor) from hospitals remained uncollected in Deva with COVID-19 patients [35].

Such wastes should be temporarily stored in special locations maximum 24 h unless the wastes are stored in a location provided with a cooling system that constantly ensures a temperature of less than 4 °C, a situation in which the storage period can be a maximum of 7 days according to technical norms of Ministry of Health [36].

Ministry of Environment, Water and Forests emitted an order during the emergency state in Romania (2 April 2020) that all public land should be cleared by illegal dumping sites until 30 May 2020. These illegal dumping sites are considered by environmental authorities as an additional factor for the spreading of the coronavirus. Previous controls made by the National Environmental Guard in the first trimester of this year detected several wild dumpsites. Local authorities are responsible to collect and properly dispose of such wastes. Uncontrolled waste disposal practice is a serious environmental issue in Romania where peri-urban and rural regions are the most exposed to such practices [15]. Each spring season, the local authorities must perform sanitation activities around their localities. Seasonal floods feed freshwater pollution of dam lakes or downstream localities. Plastic pollution reached alarming levels in Romania due to waste mismanagement activities [14]. By the end of alert state 15 June 2020, local authorities of Bals city cope with massive plastic pollution and wood wastes carried out by Oltet river from upstream localities due to the summer flash floods.

Sorting stations, where dry recyclables are manually sorted, are an additional source of contamination for workers. Some household items that can be included in the category of infectious waste (napkins, masks, gloves, cutlery, containers, etc.) should not be separately collected, but to be disposed of in the residual bins. Some sorting lines were stopped to avoid the contamination risk of workers due to the presence of such items in recyclables bins. Environmental authorities detected (during March) massive illegal burning practices of hazardous and nonhazardous waste items (e.g., used tires, electric cables) in Ilfov County (Sintesti village of Vidra commune) which polluted the air of Bucharest city. The circular economy is limited in Romania where landfill is still the most prevalent option in case of municipal waste stream despite the most ambitious plan supported by the EU [17].

Medical waste management in Romania is less studied than the municipal waste stream and available data are scarce. However, some studies reveal that improper management of medical wastes can lead to water pollution [37] and other environmental threats [38]. Before the EU accession, the medical waste flow was disposed of in old and improper crematories of hospitals [39]. Despite some early guidelines on healthcare waste management practices [40] the problem of this waste stream is far to be solved across the EU. There is no Europe wide overview of amounts of unused pharmaceuticals and their return rate which poses further risks to the environment [41].

**Figure 5:**
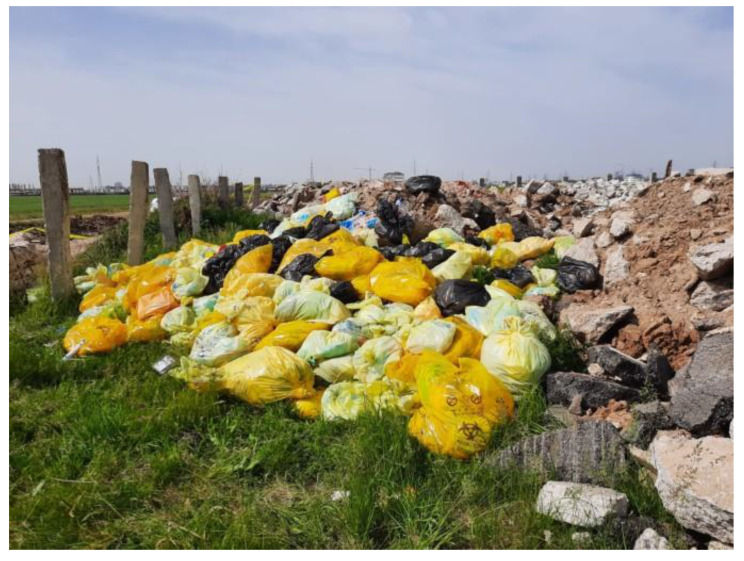
Illegal dumping of medical waste in Dobroiesti commune, Ilfov County. 14 May 2020 (source: National Environmental Guard).

Environmental authorities made field controls on 14 May about the illegal waste disposal of hazardous medical wastes collected from Bucharest hospitals on open dumps in rural areas of Ilfov County as shown in Figure 5. These wastes were collected and transported by a special waste operator under the supervision of environmental authorities.

The problem of pharmaceutical waste-related issues gains attention in recent studies which point out the necessity of cohesion between legislative framework, waste management infrastructure, public policies and awareness campaigns [42,43]. Medical waste disposal is provided also by hazardous waste incinerators. However, hazardous waste incinerators are a controversial topic in the environmental policies of Romania.

In Dolj County, the medical waste incinerator which operates in Sopot commune was fined by the National Environmental Guard (80,000 lei) because of several non-compliances with environmental permits and measures were imposed regarding the proper storage and disposal of waste as well as related to the incinerator furnaces parameters [44].

In Suceava County, the environmental authorities found 1019.8 kg of hazardous medical waste improperly stored in a hall. In 2018, 11 hazardous waste incinerators (which also dispose of the medical waste), 14 treatment facilities (thermal decontamination at low temperatures) and 23 on-site treatment facilities (hospitals) operated in Romania [45]. According to the Ministry of Environment, there are 9 hazardous waste incinerators in Romania which operate in 2020 which can burn 100–500 kg of medical waste per hour and 15 medical waste sterilization facilities which can treat 45 t of medical waste per day in the COVID-19 pandemic context [46].The national waste management plan point out the small number of thermal decontamination treatment facilities at low temperatures of hazardous medical waste (inside the sanitary units or in a centralized system). In Romania, there are 14 counties with no such treatment facilities increasing dependency on hazardous waste incinerators as alternatives for waste disposal options [18]. Environmental NGO’s request national authorities to stop medical and hazardous waste incineration in facilities located near human settlements during the emergency state in Romania because air pollution could increase the COVID-19 cases, particularly in large cities [47]. These organizations draw attention on several environmental concerns such as illegal activities related to recent waste burning practices must be investigated by environmental authorities; to stop the import of any type of waste or second–hand goods; to speed up the acquisition process of monitoring equipment in case of dioxins, furans and PCBs and made that data publicly available.

### 3.4. Best Practices During the Emergency State Conditions

The Ministry of Health, National Institute of Public Health and the Ministry of Environment, Water and Forests released a notification for the general public regarding the management of municipal waste stream which transposes the UNEP and ECDC guidelines on this matter: (i) municipal waste generated in households with confirmed/suspected COVID-19 cases must be disposed of in the residual bin (without separate collection), the bag must be carefully enclosed without any compression, closed access to animal companions near the waste bags/bins, these items will be collected by municipal waste operators and directed transported to landfills; (ii) municipal waste generated in households without confirmed/suspected cases will be managed as usual with proper separate collection schemes. Clear communication with the general public is an essential step towards sound waste management practices of potential infectious wastes during the COVID-19 pandemic.

The National Institute of Public Health declared household waste generated in quarantine places as infectious wastes (18 March 2020), therefore, a strict waste management procedure must be enforced in this regard. These wastes must be collected by special waste operators and transported at −4 °C to hazardous waste incinerators. Waste operators and/or inter-community development associations that supervise waste management activities at regional level release guidelines with specific requirements on how to handle municipal waste stream in case of confirmed, quarantine or self-isolated citizens [48].

**Figure 6:**
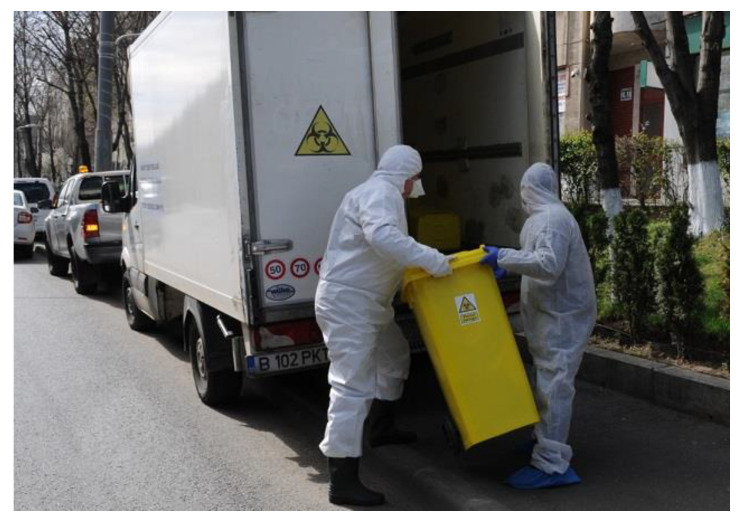
Collection of COVID-19 infectious waste in District 4 of Bucharest city Source: Bucharest District Hall 4.

In Bucharest city, local authorities of District 4 and 6 provide special bags for waste collection of quarantine places and from self-isolated persons. District 4 bought two special vehicles (capacity of 840 L each, seven bins of 120 L) dedicated to collect and transport potential infectious wastes at −4 °C to waste incinerators from Dolj and Prahova counties [49]. These vehicles will cover the quarantine site and self-isolated persons with a twice collection frequency per week in District 4 of Bucharest city. The workers have full protection equipment as shown in Figure 6.

Waste workers equipped with PEE equipment are compulsory and they must have access to daily disinfectants. In Neamt County, waste operators changed the waste collection frequency of dry recyclables to once per month in rural areas. In addition, the dry recyclables bags must be enclosed and have indicated by the generator on the date of closing the bag. However, the reduction of waste collection frequency in rural areas can lead to illegal dumping or open burning practices [50].

Optimized routes for medical waste collection at regional levels are necessary to avoid additional risks in the context of COVID-19 pandemic when hospitals generate higher amounts of medical waste and there is increasing pressure on hazardous waste transporters and waste incinerators [51]. During the peak of COVID-19 pandemic, storage and waste disposal facilities are surpassed by the huge amounts of medical wastes in countries like in China and Italy [32,52].

The COVID-19 pandemic reveals that medical waste management treatment facilities must be further developed and to decrease the dependency on hazardous waste incinerators. Civil society and mass-media reveal several serious issues related to public health and environmental pollution of waste incineration plants in Romania in the last years. This waste disposal option is a critical environmental concern around the world [53].

On-site treatment facilities (hospitals) can be cost-efficient compared to the collection, transportation and incineration costs [32,54].

The National Police, National Environmental Guard and Health Inspection performed 283 controls (from 9 June to 1 July) at the sanitary units and at the economic agents that carried out operations in the field of hazardous waste management infested with SARS-CoV-2 virus. This operation led to safely disposing of 210 t hazardous waste, 43 fines (amounting to 454,800 lei) 40 warnings, 6 criminal cases and temporary closure of an incinerator [55].

Alternatives options to waste incineration is mandatory in the medium term in Romania such as [56]: (i) thermal processes at low temperature (105 °C–177 °C) using hot air inactivation equipment, steam equipment, microwave inactivation equipment or inactivation equipment by crushing, steaming, drying process; (ii) chemical processes—medical waste is first crushed and mixed to increase its exposure to the action of chemicals (sodium hypochlorite, peracetic acid or inorganic chemicals) and the disinfectant solution is recycled; (iii) Irradiation-based technologies refer to the exposure of medical waste to the action of electronic particles, cobalt-60 rays or UV rays; (iv) biologic processes use enzymes to break down organic matter. In Romania, the thermal process at low temperatures is the most widespread medical waste treatment as an alternative to waste incineration plants. However, only 6% of healthcare facilities investigated in 2018 (out of 832) treat medical waste in its facilities (thermal process at low temperature) and 15% had contracts with such specialized economic agents [45]. Most healthcare facilities (65%) are reliant on waste operators which transport medical waste flow to hazardous waste incinerators while 14% dispose of their nonhazardous waste through landfilling [45]. Improvements in staff awareness related to proper medical waste management practices and green purchasing suppliers are further steps in reducing pollution threats [57]. Contingency plans to target waste management sector under various scenarios should be continuously developed and adjusted [58].

## 4. Further Recommendations

Based on medical and municipal waste management issues raised by coronavirus spreading in Romania (Section 3), a set of recommendations is proposed at the EU and Romania levels to better monitor the COVID-19 waste flow, municipal, and medical waste stream in general, as a key support for decision-makers.

### 4.1. EU Commission

Policy and monitoring issues must be addressed and improved among EU countries under the supervision of the EU Commission to provide reliable data about waste flows during the COVID-19 pandemic in short and medium-term such as:

- Special reports about waste management challenges in both medical and municipal waste management sectors associated with COVID-19 pandemic;
- Eurostat must include medical waste management statistics as a compulsory special waste stream broken down by waste items (infectious waste included) beside the existing ones (e.g.WEEE, etc.);
- Infectious-waste generation rates (kg∙bed∙day^−1^) and other similar medical waste flows must be determined by experimental studies across EU countries.

In the long term, waste management policies should have the following key objectives:

- The European Commission must include the issues of healthcare waste management activities in annual environmental reports and healthcare performance status with separate case reports for each country;
- Comprehensive health and waste-related statistics and common policies to cope with future outbreaks;
- Circular economy policies must include the medical waste management sector with clear guidelines and best practices;
- EU funds to develop and support sound medical waste treatment facilities as an alternative to hazardous waste incineration plants.

### 4.2. Romania

Poor publicly available data related to medical and municipal waste management flows was the norm before the COVID-19 pandemic in Romania, particularly at the county level, which makes it more difficult to assess the COVID-19 related waste flow in such time of crisis.

This situation was worsening during the emergency state due to the lack of COVID-19 related data at subnational levels as pointed out in Section 2 coupled with waste management deficiencies as highlighted in Section 3. Therefore, in the short and medium-term, national authorities should improve the transparency of key data:

- Centralized daily COVID-19 statistics available at county levels including a wider range of indicators (as requested by Geo-spatial.org [26]) with open access data;
- Publicly available data about environmental crimes detected by National Environmental Guard regarding waste collection and disposal (incineration/landfilling) of hazardous waste including medical waste flow;
- Intensive controls of incinerator’s operations, hazardous waste collection, open burning and illegal dumping activities during COVID-19 pandemic supervised by special environmental commissions with inter-institutional members;
- Special reports on such environmental crimes with available data at national and county levels for general public and mass-media;
- Regional public health and environmental authorities must cooperate to provide reliable monitoring of hospitals with COVID-19 patients in terms of possible environmental and public health threats associated with medical waste mismanagement practices;
- Incinerators and waste operators dealing with hazardous waste (including medical waste fractions) should mandatorily publish on their websites: environmental authorization, annual environmental performance report with detailed statistics about the wastes collected, treated and disposed of. Monthly reports of medical waste collected and treated during the COVID-19 pandemic;
- Medical-waste composition data and generation rates supported by experimental studies in each county with special attention to COVID-19 hospitals.

To enhance environmental policy decisions at national and regional levels in a post-COVID-19 pandemic context and to be better prepared for other possible outbreaks, the public access to basic waste related data must be improved:

- Regional and local waste management plans must have a special section of medical waste management systems with updated data;
- Environmental reports (national and regional levels) must include an updated analysis of medical waste management status in collaboration with the National Public Health Institute;
- Special regulations/amendments in reporting waste statistics data and to be available to the general public in electronic format (open data);
- Waste statistics should be available on national and subnational levels including NUTS3 regions of Romania;
- Waste statistics portal in web GIS format with open data available at the national, county and local administrative levels (LAU-2—cities and communes).

Investments to support alternative and environmentally friendly medical waste treatment facilities to waste incineration plants must be a continual effort in Romania.

## 5. Conclusions

This paper provides a rapid assessment of potential COVID-19 medical waste flow in Romania during the emergency state (15 March to 14 May) and alert state (15 May to 15 June) at the national level and some regional statistics. This flow is feed by confirmed COVID-19 cases, people in quarantine places, and those suspected and self-isolated in households.

The model estimates a total COVID-19 related flow of 4312 t at the national level from 25 February to 15 June of which 2633 t in the emergency state period.

The self-isolation source dominates in this waste flow for each period analyzed, but this waste stream has the lowest potential infectious risks compared to those generated by healthcare facilities (confirmed COVID-19 patients) and quarantine places. This waste flow is often mixed collected by municipal waste operators and disposed of through landfills. Both healthcare and quarantine wastes are considered infectious wastes by national authorities, therefore, around 1008 t should have been collected by special hazardous waste operators and disposed of in the waste incineration plants or using alternative options for medical waste flow as outlined in Section 3.4. The paper reveals a shift between quarantine and medical wastes in the alert state, where the estimated amounts of waste generated by confirmed COVID-19 patients in healthcare facilities (167.47 t) are larger than those in quarantine places (94.51 t).

There are regional disparities regarding the amounts of infectious waste (healthcare + quarantine sources) compared to self-isolation wastes among Romanian counties as shown by the map (Figure 3), but the capital city of Bucharest is the largest contributor to the overall COVID-19 related waste flow and self-isolation waste on this regional snapshot (4 April). Furthermore, Suceava County is the largest contributor to medical waste and Constanta County for quarantine wastes.

The COVID-19 waste flow cannot be determined for all periods at subnational levels due to the limited data. However, the Neamt County (one of the top five counties affected by COVID-19) is examined as a case study of local COVID-19 related waste flow during the emergency state.

The paper points out several critical issues related to medical and municipal waste management sectors in Romania. Monitoring of COVID-19 waste flow through the proposed model is important for decision-makers, particularly in low and middle-income countries like Romania which are facing waste management deficiencies and gaps in waste statistics, to reduce other contamination risks or related environmental threats. The regional analysis (subnational levels) of COVID-19 related waste flow provided by spatial statistics and thematic cartography is crucial in this regard, but more transparency and public access to such COVID-19 related data are required from national authorities.

The mentioned recommendations would enhance better decisions across EU and Romania in mitigating the waste-related secondary impacts on public health status and environmental factors during such biologic hazards or other possible hazards (natural or anthropogenic) caused by climate change or environmental pollution.

Both medical and municipal waste management services are critical around the world and these systems must cope with serious challenges particularly in low and middle-income countries. Further investigations and research studies are necessary to adjust the COVID-19 waste-related flows in Romania and other countries affected at considerable levels by this pandemic.

## Figures and Tables

**Table 1:** COVID-19 waste flow at the national level from 26 February to 15 June 2020.

Period / Tons Generated	ICU Patients Related Waste	Medical Waste COVID-19	Quarantine Waste	Self-Isolation Waste	Total COVID-19 Related Waste
Prior emergency state (26 February–15 March)	0	0.44	5.79	109.72	115.96
Emergency state (16 March–14 May)	17.17	286.04	453.44	1893.56	2633.05
Alert state (15 May–15 June)	10.1	167.47	94.51	1301.81	1563.79
Total period (26 February–15 June)	27.28	453.95	553.75	3305.1	4312.81
